# The Incidence of Road Traffic Crashes Among Young People Aged 15–20 Years: Differences in Behavior, Lifestyle and Sociodemographic Indices in the Galilee and the Golan

**DOI:** 10.3389/fpubh.2018.00202

**Published:** 2018-07-26

**Authors:** Shai S. Klaitman, Evgeny Solomonov, Amir Yaloz, Seema Biswas

**Affiliations:** ^1^Department of Surgery, Ziv Medical Center, Bar Ilan Medical School in Safed, Safed, Israel; ^2^Department of Surgery, Ziv Medical Center, Safed, Israel; ^3^Upper Galilee and Golan Heights, National Road Safety Authority, Jerusalem, Israel; ^4^Department of Surgery, Ziv Medical Center, Safed, Israel

**Keywords:** road traffic crashes, childhood injuries, socioeconomic inequalities, risk behavior, young drivers

## Abstract

**Background:** Adolescent injuries and fatalities constitute a world health concern and present a major public health burden. Of all childhood injuries in Israel 61.8% occur during road traffic crashes. The risk of being injured or killed in a road traffic crash is especially high among individuals deemed to have a low socio-economic status, but, there also exist crucial risk factors for road traffic crashes that are intrinsic social determinants of health, including behavior, lifestyle, education and employment. These associations are explored in this study.

**Aim:** To examine the contribution of socioeconomic inequalities to the incidence of road traffic crashes among adolescents living in the Galilee and the Golan.

**Methods:** A large retrospective study of trauma records (*N* = 3293) of 15–20-year-old patients who were injured in road traffic crashes and admitted to Ziv Medical Center between 2004 and 2015 was performed. These patients were subcategorized according to their home address. Using census data and other databases associations between socioeconomic inequalities, intrinsic social determinants and the prevalence of road crashes were investigated.

**Results:** Road traffic crashes in the Galilee and the Golan are more frequent among residents of low socioeconomic areas. Fifty-five percent of drivers are from the areas of lowest socioeconomic level (levels 2 to 4) and are responsible for 60% of the road traffic injuries admitted to Ziv Medical Center (*p* < 0.001). In contrast, 42% of drivers are from areas of medium socioeconomic level (levels 5 and 6) but are responsible for only 35% of road traffic injuries (*p* < 0.001). Young women in the Galilee and the Golan are less likely to be involved in road traffic crashes−35.8% compared to 64.2% in young men.

**Conclusions:** This research has shown that youth, male gender, socioeconomic status, education level and the quality of road infrastructure are important factors in the incidence of road traffic crashes in the Galilee and the Golan. This should be taken into consideration by policy makers in order to develop appropriate interventions in road safety targeted at multiple levels.

## Background

Injuries are a major public health problem worldwide and constitute the leading cause of death among adolescents in developed countries (1). Nearly 90% of injuries to adolescents are the result of unintentional or “accidental” incidents (2). Worldwide, pedestrian vehicle crashes are the second greatest cause of child mortality (3). Beterem, an organization which aims to promote child safety in Israel, reported in 2008 that 61.8% of childhood injuries are due to road traffic crashes (4). According to Israel's Central Bureau of Statistics, in 2015, there were 23,488 casualties from 12,661 road traffic crashes (5), of whom 848 were adolescents brought to Ziv Medical Center, in Safed, Galilee, for treatment.

### Socioeconomic status as a determinant of road traffic injury

There is increasing focus on the effects of socioeconomic status as a determinant of health among both individuals and communities, however, relatively few studies have focused on the influence of socioeconomic status on road-traffic crashes (6). Studies show that drivers of lower socioeconomic status have a higher risk of fatal and non-fatal traffic-related injuries (including motor vehicle, pedestrian, and bicycle injuries) (6–8). Drivers from lower socioeconomic areas are almost twice as likely to be involved in a collision than drivers from high socioeconomic areas (9). A number of explanations have been proposed: longer distances traveled (as a result of living in the “periphery” far from urban centers of work) (7), driving cars that are less road worthy, and shared risk behaviors such as failure to wear seatbelts, the use of mobile telephones while driving, and ignoring road traffic signs (10).

Some studies, on the other hand, have failed to identify a statistically significant correlation between socioeconomic status and traffic incidents. Faelker et al. (11) examined the socioeconomic differences in childhood injury in Ontario, Canada. Their results showed a positive correlation between lower socioeconomic areas and injuries resulting from falls, injuries in the home, and during recreation activities. The results were almost statistically significant (*p* = 0.06) for the correlation between impoverished areas and the risk of traffic injuries and injuries of extreme severity but they studied only 400 incidences of road traffic injury (dividing these 400 into five subgroups based on level of income). These limited numbers within subgroup analysis (*n* = 31, for example) failed to show significant correlation between injury and socioeconomic level.

Another study by Anderson et al. (12) examined the relation between the socioeconomic status and injury in adolescents and found that socioeconomic status does not seem to be a contributing factor to injury. These findings are contrary to the prevailing literature citing associations between socioeconomic status and injury. Anderson et al studied a cohort of teenagers (aged 12–16 years) from a single school. The teenagers had homes in eleven communities within the school district. Students were from various socioeconomic backgrounds (evaluated by the researchers on the basis of student descriptions of their parents' work—specifically, whether both parents were in employment). The total student population across all ages was 1,245 (high socioeconomic level *n* = 337, middle *n* = 462, low = 446). Thus, Anderson et al.'s methodology included comparison between heterogonous samples and subjective analysis of socioeconomic status.

Lastly, Wise et al. (13) sought to elucidate socioeconomic disparities in mortality from birth to adolescence in Boston, United States of America. Wise et al. showed that during adolescence 57% of all deaths were the result of motor vehicles and homicide. Contrary to several studies referenced in their paper, Wise et al.'s data from Boston showed that in young people under the age of 20 years, as socioeconomic level increased, mortality due to road traffic crashes increased. This finding was explained by the extensive use of Boston's major public transport system by low income families in the urban center, leaving the occupancy rate of private vehicles to wealthier young adults living further from the city center in areas where driving oneself is essential for transportation. This introduces an important argument for the implementation of public policies that support transportation networks that address differential mortality rates among the youth. All these studies support the case for further research into the effects of socioeconomic status on the likelihood of being involved in a car crash.

### Youth as a factor in road traffic crashes

Young drivers are over-represented in road traffic injury and mortality statistics in most European countries, Australia and the USA (14). Thus, road traffic crash prevention in adolescent and young drivers is a key concern. The National Road Safety Authority of Israel found that among young people road traffic injury constitutes 34% of all trauma−15% more than any other cause of trauma in this age group (15). These alarming trauma statistics form the basis of this research–investigating road traffic injuries in the Galilee and the Golan in the 15–20-year age group.

### Road traffic crashes in Israel

Israel is a high-income country. A large proportion of Israeli towns, however, are similar to low- and middle-income countries in terms of their infrastructure and the lifestyle of their residents. These towns are characterized by poor education, employment and transport infrastructures (7, 16). There are also important deficiencies in these towns in the provision of important public safety measures such as street lighting, pedestrian crossings, stop lines, road markings and speed bumps.

In 2004, the National Insurance Institute of Israel (NII) (17) reported that in the center of Israel, 8.8% of adults and 7.1% of children live in poverty. In Galilee, however, the percentage is as high as 30.2% of adults and 41.6% of children, respectively (17). In terms of public health, indices in the center of Israel are similar to that of other industrialized western countries (low infant mortality, cancer, diabetes, heart disease) (18). Some parts of Galilee are akin to the developing world in terms of infant mortality and the incidence of infectious diseases, such as brucellosis and hepatitis (16). For this reason, an investigation of the effects of socioeconomic status on the incidence of road traffic injuries among children and young adults in the Galilee and Golan regions of Israel is of particular importance.

Injury prevention in children and young adults should be based on an understanding of socioeconomic status and epidemiological patterns of injury (19). In this research we examine predictor variables of age and gender involvement in road traffic crash. Inevitably, this involves an analysis of the social determinants of injury and safety. Where these are identified they are further explored.

## Materials and methods

### Patients: data from Ziv medical center

With hospital ethics committee approval, retrospective data were collected on all patients aged 15–20 years injured in road traffic crashes from 2004 to 2015. For each patient the following data were recorded: age, sex and postal code of the town of residence. Patients with home addresses outside the catchment area of Ziv Medical Center were excluded. Data were collected from hospital electronic patient records and stored in a password protected file accessed only by the researcher and supervisor. Patients included drivers, passengers and pedestrians.

#### Age group analyzed

The age range of 15–20 years was chosen in order to include teenagers and young drivers shown by the Israel National Road Safety Authority (20), to be prone to a higher rate of road traffic crashes.

At the age of 15 and a half years, teenagers in Israel may apply for a motorcycle driving license which they may receive on their 16th birthday. A motor vehicle driving license is issued upon passing a written theory test and a practical on-road driving test. The minimum age requirement for this is 16.9 years. Globally, motorcycles are implicated in a higher incidence of road traffic crashes (21).

### Demographic data: data from national data bases

Using the Central Bureau of Statistics of Israel database (22), the socioeconomic level of the towns of residence of the patients was recorded. Also recorded were the number of people in each town with a driving license, the number of cars registered to the town of residence, and the age of the car.

### Associations of road traffic crashes in the Galilee and Golan with socioeconomic status

Socioeconomic status in Israel is determined by the Central Bureau of Statistics and is a function of 16 different variables collating aspects of demography, education, employment and standard of living into a single index. All localities in Israel are classified into one of ten categories of socioeconomic status, one being the lowest and ten being the highest. In order to analyze trends in road traffic crashes in relation to the socioeconomic level of the town in which each patient lives, all towns within the catchment area of Ziv Medical Center were assigned to three socioeconomic groups (SES): low socioeconomic levels (2–4), medium levels (5–6), and high levels (7–8). For each town in the three socioeconomic groups, census data (22) was used to establish the number of inhabitants with driving licenses and vehicles (**Table 2**).

#### Data on the 12 towns with the most road traffic crashes

Twelve towns within the Ziv Medical Center catchment area in the Galilee and the Golan were found to have a higher number of casualties than other areas (**Figure 2**). Demographic and socioeconomic data for these towns (including the number of residents, number of high school graduates, university graduates, and salaries of the residents) were taken from the Central Bureau of Statistics database (2015) (22, 23). These data were cross-checked and verified with other national data resources from the Or Yarok Association for Safe Driving in Israel and Public Use Files from the Central Bureau of Statistics data on road safety in Israel. (24–27).

#### Investigating associations and risk factors for road traffic crashes in the 12 towns

Patients from these 12 towns in the Galilee and the Golan were identified from hospital records of their home addresses. Although the road traffic crash may not have taken place in their home town, national road traffic crash data from the National Road Safety Authority (20) lists crash data according to the home addresses of crash victims, making associations between crash data and socioeconomic characteristics of the towns from which patients originate theoretically possible. Associations were, therefore, researched between these towns in the Galilee and the Golan and risk factors for road traffic crashes. The factors included: the site of the crash—urban or non-urban, whether the crash occurred during the day or night, the roads where crashes occurred, and the nature of the crash (pedestrian or single or multi-vehicle).

### Statistical analysis

One sample *t-*test analysis was used to test the differences between study groups for distribution of driving licenses, vehicles ownership and hospital admission after car crash according to socioeconomic level, gender and kilometers travelled. A *p*-value of 5% or less was considered statistically significant. The data were analyzed using Microsoft EXCEL for Mac version 15.7 and SPSS version 23 (SPSS Inc., Chicago, IL, USA).

## Results

### Data of all patients presenting to Ziv medical center

**Figure 1:**
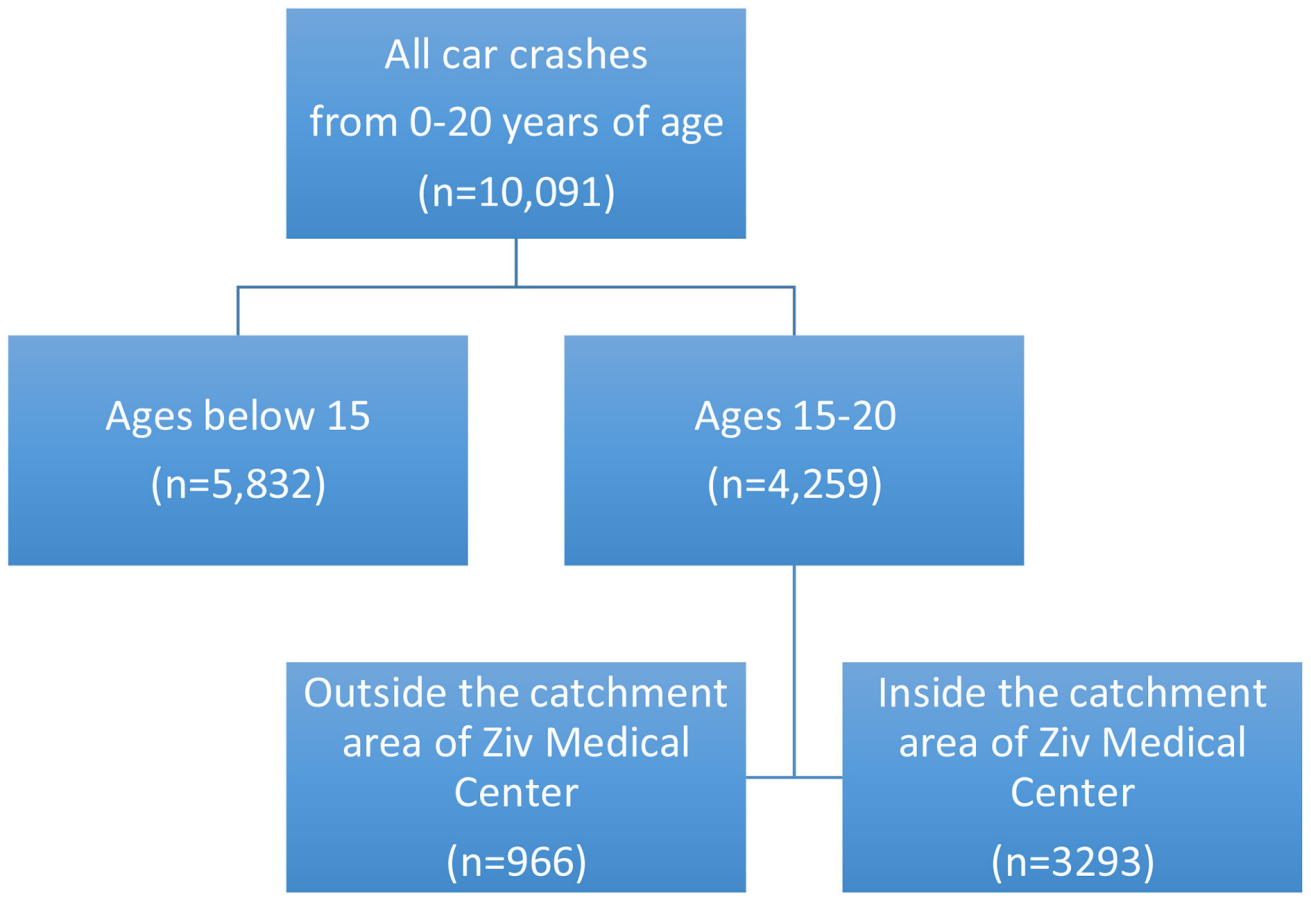
Sample Selection.

A total of 10,091 patients presented to Ziv Medical Center with road traffic crash injuries between 2004 and 2015. Of these 4,259 were within the age range of 15 to 20 years. Of these 3,293 had home addresses within the Ziv Medical Center catchment area (Figure 1).

Patient data for all 3,293 patients presenting to Ziv Medical Center with road traffic injuries from 2004 to 2015 are summarized in Table 1.

**Table 1:** Patient characteristics.

**Variable**	**Number of patients (*n*)**	**Percentage of patients (%)**
**SEX**		
Male	2,113	64.2
Female	1,180	35.8
Total	3,293	
**AGE**		
15	220	6.68
16	308	9.35
17	541	16.42
18	762	23.1
19	896	27.20
20	566	17.18

According to Central Bureau of Statistics data, towns with low SES (2–4) account for 54.4% of driving licenses and 56.9% of vehicles, towns with medium SES (5–6) for 41.6% of driving licenses and 38.9% of vehicles, and towns with high SES (7–8) for 4.1% of driving licenses and 4.2% of vehicles. Confidence intervals (Table 2) give interval estimates of percentage hospitalizations from towns of each SES. For example, there is a 95% probability that the proportion of hospitalizations from low SES towns is between 58.5 and 61.9%. Hospitalizations due to car crashes are significantly more likely among patients from towns of low SES (*p* < 0.001), while towns with medium SES account for a smaller proportion of hospitalizations than we might expect if SES and hospitalizations are independent of each other (*p* < 0.001). The contribution of towns with the highest SES is not significantly different from the corresponding percentage of driving licenses and vehicle ownership in those towns (*p* > 0.05).

**Table 2:** Socioeconomic level, vehicle license holders and vehicle owners in towns with patients admitted to Ziv Medical Center after car crash.

**Socioeconomic level of towns (22)**	**Percentage of driver's license holders in each town (%) (22)**	**Percentage of vehicle ownership in each town (%) (22)**	**Admissions to Ziv Medical Center after car crash (95% CI)**
Low	54.4	56.9	60.2 (58.5–61.9)
Medium	41.6	38.9	35.0 (33.3–36.6)
High	4.1	4.2	4.8 (4.1–5.5)

*CI, Confidence Interval*.

Further, in the case that SES significantly impacts the incidence of car crash, statistical differences emerge between each of the three SES groups in the incidence of hospitalizations. Comparison was, therefore, made between the sample percentage of hospitalizations and hypothesized values based on percentages of driving license holders and vehicle ownership among residents of each town, using Central Bureau of Statistics data, using a one-sample *t*-test. Table 2 shows the results of testing the null hypothesis (H0):{Percentage of hospitalizations after car crash = driver's license holders in the towns} vs. the alternative hypothesis (H1): {Percentage of hospitalizations after car crash ≠ driver's license holders in the towns}. Thus, the percentage of hospitalizations after car crashes in our sample (60.2%) is significantly higher than the 54.4% predicted when SES is not taken into account [t_(_3, 291) = 6.8054, *p* < 0.001].

### The role of gender in road traffic crashes

Tables 3.1, 3.2 show the number of injuries due to road traffic crashes in the 15–20 and 17–20 year age groups with regard to gender for all towns in the catchment area of Ziv Medical Center. Figures are taken from hospital data, from the Central Bureau of Statistics report about gender differences across a range of parameters (demographics, health, occupation, income, crime, and driving) (25), and the National Report of Trauma injuries in Israel (28).

**Table 3.1:** Gender distribution of drivers and hospital admissions after car crash (age 15–20).

**Gender**	**Drivers in the population in Israel (%) (25)**	**Non-car crash trauma in Israel 2009 (%) (28)**	**Admissions to Ziv Medical Center after car crash (95% CI)**
Male	58	60.3	64.2% (62.5–65.8)
Female	42	39.7	35.8% (34.2–37.5)

*CI, Confidence Interval*.

**Table 3.2:** Gender distribution of drivers and hospital admissions after car crash (age 17–20).

**Gender**	**Drivers in the population in Israel (%) (25)**	**Non-car crash trauma in Israel 2009 (%) (28)**	**Admissions to Ziv Medical Center after car crash (95% CI)**
Male	58	60.3	65.4% (63.7–67.2)
Female	42	39.7	34.6% (32.8–36.3)

*CI, Confidence Interval*.

In order to examine the impact of gender on the incidence of car crash, comparison was made between the sample percentage of hospitalizations and hypothesized values based on the percentages of drivers in the population of Israel and trauma not caused by car crash in Israel, using Central Bureau of Statistics data and a one-sample *t*-test. Tables 3.1, 3.2 show the results of testing the null hypothesis (H0):{Percentage of hospitalizations after car crash = Drivers in the population of Israel} versus the alternative hypothesis (H1): {Percentage of hospitalizations after car crash ≠ Drivers in the population of Israel}. Thus, as 58% of the Israeli population driving license holders are male, we would expect them to account for 58% of hospital admissions after car crashes, but instead they represent 64.2% of admissions [*t*_(3, 291)_ = 7.3638, *p* < 0.001].

### Data of the 12 towns with the most casualties presenting to Ziv medical center

**Figure 2:**
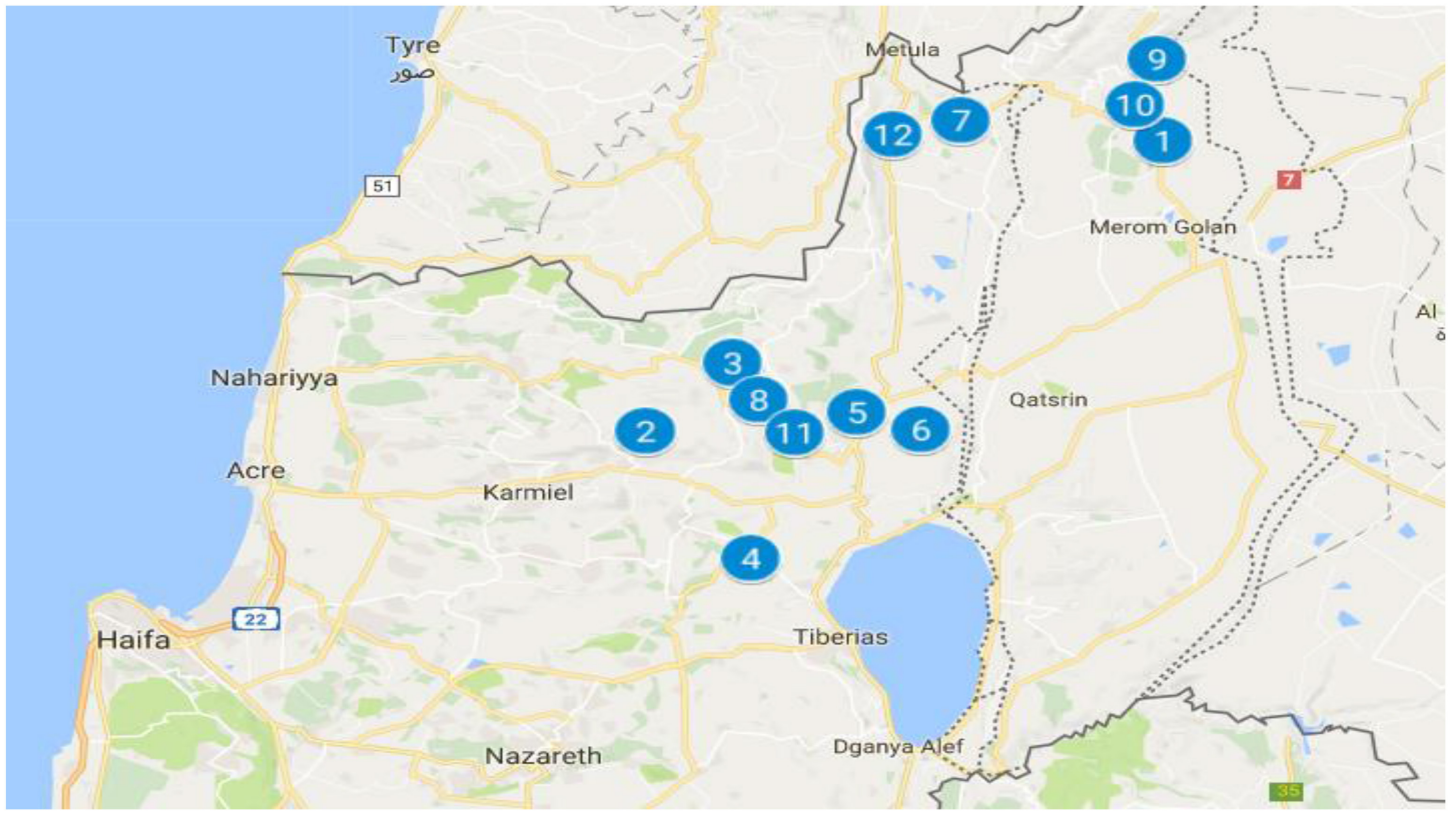
Twelve towns in the Ziv Medical Center catchment area with the most road traffic crash casualties (Google Maps, 2017)1- Buq'ata; 2-Beit Jann; 3-Jish; 4-Mughar; 5-Hatzor HaGlilit; 6-Tuba-Zangariyye; 7-Upper Galilee Regional Council; 8-Merom HaGalil Regional council; 9-Majdal Shams; 10-Mas'ade; 11-Safed; 12-Kiryat Shmona.

Twelve areas within the catchment area of Ziv Medical Center had, both according to absolute numbers of patients and by proportion of the population of the towns, the largest numbers of casualties admitted to Ziv Medical Center (Figure 2).

Table 4 summarizes the characteristics of the 12 towns with the most road traffic crash casualties within the Ziv Medical Center catchment area.

**Table 4:** The 12 towns in the Ziv Medical Center catchment area with the most patients involved in car crashes.

**Town**	**Socioeconomic level (22)**	**Population size (22)**	**Number of motor vehicle driving license holders in town (2014) (22)**	**Number of vehicles registered to town (2014) (22)**	**Number of car crashes after which patients were treated in Ziv Medical Center (2004-2015)**
Majdal Shams	2	10,643	4,902	4,058	189
Buq'ata	2	6,329	2,053	1,969	162
Beit Jann	2	11,426	4,397	3,362	122
Tuba-Zangariyye	2	6,270	2,441	1,808	120
Mughar	2	21,650	8,423	1,808	106
Mas'ade	2	3,520	1,542	1,427	82
Hatzor HaGlilit	3	8,834	4,131	2,875	185
Safed	4	33,358	11,683	7,202	649
Kiryat Shmona	5	23,076	11,829	7,802	391
Merom HaGalil Regional Council	5	12,933	6,750	5,310	254
Jish	5	3,078	1,638	1,213	69
Upper Galilee Regional Council	6	14,765	8,518	5,170	121
Total		*n* = 155,882	*n* = 68,307	*n* = 48,572	*n* = 2450

Table 5 summarizes the characteristics of the car crashes that residents of the 12 towns within the Ziv Medical Center catchment area were involved in between 2014 and 2016.

**Table 5:** Car crash characteristics (26) for residents of the 12 towns with most patients presenting to Ziv Medical Center.

**Town**	**Pedestrian**	**One vehicle crashes**	**Two vehicles crashes**	**Two wheelers**	**Crashes in non-urban areas**	**Crashes in urban area (residential)**	**Crashes in urban area (non-residential)**	SES^*^
Majdal Shams	2	8	13	1	84	19	26	2
Buq'ata	2	6	22	3	78	18	16	2
Beit Jann	2	9	15	5	113	39	17	2
Tuba-Zangariyye	2	11	10	1	55	5	15	2
Mughar	3	13	49	4	207	77	58	2
Mas'ade	2	4	2	6	31	18	9	2
Hatzor HaGlilit	2	2	7	6	43	18	19	3
Safed	6	13	36	22	132	123	49	4
Kiryat Shmona	3	8	28	18	97	82	23	5
Merom HaGalil Regional Council	4	9	4	5	87	18	37	5
Jish	1	3	6	1	38	2	10	5
Upper Galilee Regional Council	1	2	0	11	103	3	21	6

* *SES refers to socioeconomic levels according to the Central Bureau of Statistics which uses a scale from 1 to 10 where 1 is the lowest and 10 the highest. For this research towns were assigned the categories low, medium and high (low 2–4; medium 5–6; high 7–8)*.

Thus, the results show that:

- road traffic crashes in the Galilee and the Golan are more frequent among residents of low socioeconomic areas (60.2% in SES (socioeconomic levels) 2–4 compared to 35% in SES 5–6 and 4.8% in SES 7–8);
- young women in the Galilee and Golan are less likely to be involved in road traffic crashes (35.8% females compared to 64.2% in males); and
- road traffic crashes are more common among 17–20-year-old youths than 15 and 16-year-olds, i.e., more likely among those in possession of a full driving license (84% versus 16%).

## Discussion

### The impact of socioeconomic status on road traffic injury

This research shows that socioeconomic level plays a significant role in road traffic injury among adolescent and young drivers in the Galilee and the Golan. Low socioeconomic status is associated with a greater incidence of road traffic injury. As inhabitants of towns may include individuals of varying degrees of wealth—this means that, individuals of higher socioeconomic status living in these areas are also at greater risk of road traffic injury as they are potentially injured by residents of lower socioeconomic status (6). Equally, individuals from these towns driving elsewhere introduce threats to road safety in other areas (Table 5). Most crashes involve vehicles rather than pedestrians. The National Road Safety Authority (20) reports that between 2014 and 2016 in the town of Jish (number #3 in Figure 2) there were only two road traffic crashes (Table 5); but drivers from Jish were involved in ten motor vehicle crashes in other towns and thirty eight crashes on inter-urban roads (roads connecting towns).

Table 2 shows that according to the Central Bureau of Statistics (22), towns classified as being of low socioeconomic level (levels 2–4) account for 54% of driving licenses and 57% of vehicles. Towns of medium socioeconomic level (levels 5 and 6) account for about 42% of licenses and 39% of vehicles, while towns with high socioeconomic level (7 and 8) account for only 4% of driving licenses and 4% of vehicles. The distribution of driving licenses and vehicles is almost identical for each socioeconomic level, therefore, one may assume that, in general, each individual in possession of a driving license owns a single vehicle.

In the areas of lowest socioeconomic level (levels 2–4) 55% of drivers were responsible for 60% of the road traffic injuries admitted to Ziv Medical Center (*p* < 0.001). In contrast, areas of medium socioeconomic levels (levels 5 and 6) 42% of drivers were responsible for only 35% of road traffic injuries (*p* < 0.001). The catchment area of Ziv Medical Hospital does not actually contain towns or villages of socioeconomic levels above 8 in the scale from 1 to 10. Indeed, there are so few areas in the catchment area of Ziv Medical Center of socioeconomic level 7 or 8 that no statistical conclusion can be drawn about the number of road traffic crashes people from these areas are involved in (*p* = 0.1077). Eight of the 12 areas (Figure 2) in the Galilee and the Golan with the highest number of inhabitants with road traffic injuries presenting to Ziv Medical Center were of socioeconomic levels 2 and 4. Further, none of these 12 areas exceeded a socioeconomic level of 6.

In terms of education, the number of students graduating from high school or achieving a university qualification was around 50% of the national average (29) in 6 of the 12 towns, and just above the national average in 4 of the 12 towns. Similarly, people without high school diplomas have been found to have a three-fold increased risk of death from road traffic crashes compared to those who completed high school (7). Magid et al confirms level of education, employment and army service as risk factors for road traffic crashes.

In general, in central Israel, the proportion of students gaining a matriculation certificate is 65.5% (10). Over the last five years there has been a cumulative increase in the rate of matriculation of 7.7% nationally and 6.6% in Majdal Shams, Buq'ata, Tuba-Zangariyye and Mas'ade (22). It will be interesting to see whether there is a proportionate increase in safe driving behaviours. The average salary of residents in the 12 towns and of car license holders in these towns is lower than the national average (22).

### The role of gender in road traffic crashes

Patients aged between 15 and 20 years from all areas within the catchment area of Ziv Medical Center comprised 64% young men and 36% young women. According to the Central Bureau of Statistics report on gender differences (25) women hold 42% of all driving licenses while men hold 58%. As not all patients presenting to Ziv Medical Center were drivers (data regarding driver or passenger status was not recorded in the original electronic patient records) a second benchmark was used to decide whether the number of male casualties was dependent on a greater proportion of male drivers or whether male gender is in itself a risk factor. Women account for 40% (28) of trauma not caused by road traffic crashes. The percentage of female patients admitted to Ziv Medical Center was only 36%; therefore, male patients had the most injuries and were involved in the most road traffic crashes.

On comparison of the female patient proportion of 36% with the population percentages of female drivers (42%) (25) and the incidence in Israel of female trauma, using the 1-sample *t*-test (Table 3.1) a statistically significant (*p* < 0.001) difference was found between rate of injury of young women versus young men. At the 95% confidence interval the true proportion of accidents involving young women is, on average, 5–8% points lower than what might be expected based on the gender distribution of drivers, and 2–5% points lower than we might be expected based on the gender distribution of trauma of all other causes. Therefore, overall, young women were found to be less often involved in road traffic crashes requiring admission to Ziv Medical Center.

The gender difference becomes more pronounced when non-license holders (15 and 16-year-old patients) are excluded (Table 4). Among 17–20-year-old patients (more likely to be drivers than 15 and 16-year-old patients who are more likely to be passengers) the advantage of young women over young men becomes even more evident as female hospitalizations account for only 35% of hospitalizations in this age group), implying that young women are safer drivers than young men.

Gender appears to be an important factor, in general, in adolescent trauma (4) and, in particular, in a young driver's risk of being involved in a crash. Crash occurrence is more pronounced among young male drivers (30) with evidence that they are inclined toward higher risk taking (31), engage in unsafe behavior (32), misjudge and overestimate their driving abilities (33), commit more traffic violations (10), drive over the speed limit (34) and are more likely to drive after smoking marijuana (32) and consumption of alcohol (35).

### Associations and risk in the physical and social environment

In terms of risk factors among young drivers in Israel, three broad categories may be discussed: crash data (36); socioeconomic characteristics of towns where drivers are from and where crashes occur; and, driver risk factors (10, 29, 35).^.^These factors inevitably interact. Suchinsky et al. (37) and Prato et al. (31) describe the increased risk of single-vehicle accidents of young drivers, multiple-vehicle accidents between young drivers, accidents involving motorcyclists or cyclists, and children and adolescents crossing roads in major urban areas (31). Residents in the 12 towns in the Galilee and the Golan analyzed in this research were more likely to be involved in inter-urban road traffic crashes (Table 5). The main inter-urban routes connecting the 12 towns, are characterised by narrow lanes and multiple intersections (route 85), roads where drivers are likely to speed (routes 90 and 65), and narrow windy roads with patchy street lighting (route 89 between Safed and Rosh Pina, and routes 70, 91, 98, and 99) (20). There were more vehicular than pedestrian crashes and more head on collisions, suggesting that speed and crossing the center line between lanes may be important behavoral factors (37).

Road infrastructure (street lighting, road width, and lanes) play a significant role in the incidence of vehicular crashes (37). A failure to invest in road infrastructure in areas of low socioeconomic status is, therefore, an important factor in crash prevention. Magid et al. (7) found that a higher incidence of road traffic crash among Israel's Arab population might be attributed to poor road safety infrastructure compared to Jewish towns. Factors included the lack of separation between roads and sidewalks, narrow roads with a lack of distinct lanes, the lack of traffic signs and lane markings, and items obscuring the driver's vision, such as walls, bushes, trash and parked cars (7). It stands to reason, therefore, that poor road safety infrastructure in all areas of the Galilee and the Golan, irrespective of ethnicity, pose a risk to all populations.

Elias et al. (38) present the town of Shefaram in Lower Galilee as a case study in the risk factors of childhood pedestrian injuries. Elias et al identified high residential density, low socio-economic status, low level urban-planning, the lack of public transportation, narrow roads and the absence of shoulders and sidewalks, the lack of distinct pedestrian crossings, the lack of traffic signs and parking facilities as risk factors in pedestrian traffic injuries. The roads where most pedestrian injuries occurred traversed towns where residential, commercial and public buildings were built around the main road passing through the town.

In Israel, the speed limit on urban roads is 50 km/h, non-urban 80 km/h, and 110 km/h on a small number of high speed roads. The speed survey conducted in Israel in 1998 of 17 inter-urban roads showed that the speed limit was exceeded by 10–96% (average 58%) compared with other OECD countries (exceeded by 15–80%).

### Using hospital and national databases to identify risk factors

**Figure 3:**
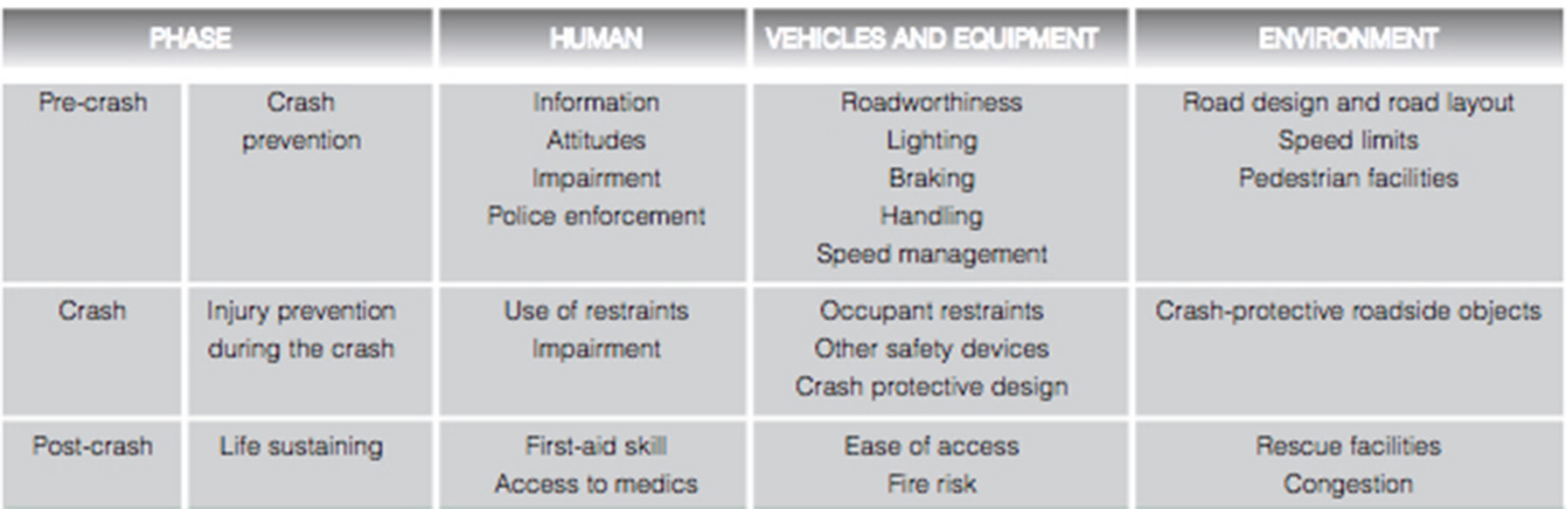
Haddon Matrix of contributing factors to injury from motor vehicle crash (39).

Figure 3 shows the Hadden-Matrix model for the analysis of risk factors that affect the outcome of road traffic crashes. This research has focussed on social and environmental factors. While it has been possible to identify risk factors that may have an important influence on the incidence of road traffic crashes, we are at pains to point out that we have not examined the outcome of road traffic crashes, nor examined injury patterns.

Our analysis has been confined largely to drawing associations between known risk factors and published population indices that we may reference to explain the incidence of crashes in the Galilee and the Golan. The hospital data had no information about the circumstances of the crash, the use of seatbelts, speed, or whether the patient was a driver or passenger.

Data from the National Road Safety Authority (26) lacked detail of where exactly on the road crashes took place (indeed much of the data recorded the name of the road as unknown, citing the lack of road names in some villages). Similarly, there was no record of traffic violations—seatbelt usage, traffic light violations and speeding. The Police database also had no statistics on the use of seatbelts, alcohol level or drug intoxication, nor a breakdown of specific traffic violations.

There is a clear need, therefore, to research injury outcomes alongside road traffic crash investigations in order to draw substantial correlations and build evidence that necessitates improved road safety and safe driving behaviors.

## Conclusion

This research has shown that youth, male gender, socioeconomic status, education level and the quality of road infrastructure are important factors in the incidence of road traffic crashes in the Galilee and the Golan.

There is a clear need to explore these associations further and to record crash details with accuracy in order to implement risk reduction strategies. Even without these details, however, there is good evidence to support policies that improve education and infrastructure in the Galilee and the Golan, and to invest in lasting road safety schemes that mitigate crashes in fast-moving traffic.

## Author contributions

SK and SB are responsible for the design and concept of the research, data retrieval and analysis. SK and SB wrote and revised the article. ES reviewed the data and revised and approved the paper. AY contributed data and data analysis.

### Conflict of interest statement

The authors declare that the research was conducted in the absence of any commercial or financial relationships that could be construed as a potential conflict of interest. The reviewer SJS and handling Editor declared their shared affiliation.
